# A Role for Eosinophils in the Intestinal Immunity against Infective *Ascaris suum* Larvae

**DOI:** 10.1371/journal.pntd.0002138

**Published:** 2013-03-21

**Authors:** Dries Masure, Johnny Vlaminck, Tao Wang, Koen Chiers, Wim Van den Broeck, Jozef Vercruysse, Peter Geldhof

**Affiliations:** 1 Department of Virology, Parasitology and Immunology, Faculty of Veterinary Medicine, Ghent University, Merelbeke, Belgium; 2 Department of Pathology, Bacteriology and Avian Diseases, Faculty of Veterinary Medicine, Ghent University, Merelbeke, Belgium; 3 Department of Morphology, Faculty of Veterinary Medicine, Ghent University, Merelbeke, Belgium; Uniformed Services University of the Health Sciences, United States of America

## Abstract

The aim of this study was to explore the mechanisms of resistance against invading *Ascaris suum* larvae in pigs. Pigs received a low dose of 100 *A. suum* eggs daily for 14 weeks. This resulted in a >99% reduction in the number of larvae that could migrate through the host after a challenge infection of 5000 *A. suum* eggs, compared to naïve pigs. Histological analysis at the site of parasite entry, i.e. the caecum, identified eosinophilia, mastocytosis and goblet cell hyperplasia. Increased local transcription levels of genes for IL5, IL13, eosinophil peroxidase and eotaxin further supported the observed eosinophil influx. Further analysis showed that eosinophils degranulated *in vitro* in response to contact with infective *Ascaris* larvae in the presence of serum from both immune and naïve animals. This effect was diminished with heat-inactivated serum, indicating a complement dependent mechanism. Furthermore, eosinophils were efficient in killing the larvae *in vitro* when incubated together with serum from immune animals, suggesting that *A. suum* specific antibodies are required for efficient elimination of the larvae. Together, these results indicate an important role for eosinophils in the intestinal defense against invading *A. suum* larvae.

## Author Summary

*Ascaris lumbricoides* and *A. suum* are common large roundworms that inhabit the small intestine in humans and pigs, respectively. Before the worms establish themselves in the small intestine, they first migrate through the host's liver and lungs, causing significant organ damage. After treatment, people and animals are quickly reinfected. An important reason for this is that immunity against this parasite is only slowly built up. In this study, we examined the intestinal immune response in animals after prolonged exposure that prevents *Ascaris* larvae from invading the host. Animals that were protected had increased numbers of eosinophils in the gut. *In vitro* assays showed that the eosinophils were able to kill larvae by releasing the toxic content of their granules after contact with the invading larvae. These findings shed new light on the mechanisms of protection against reinfections with *Ascaris*, providing a basis for further research into the development of immunological control strategies against this parasite.

## Introduction

The gastro-intestinal nematodes *Ascaris lumbricoides* and *A. suum* are amongst the most prevalent parasites of humans and pigs, respectively. Human ascariasis is a major cause of abdominal disorders in developing countries with poor sanitary conditions, especially in children [1]. In pigs, *A. suum* is responsible for important economic losses, mostly due to a worse feed conversion rate and liver condemnation [2]. In developed countries, *A. suum* is also considered a zoonotic agent [3], [4]. In addition, infection with *A. suum* reduces the efficacy of vaccines that target other pathogens, such as *Mycoplasma hypopneumoniae* [5]. Although anthelmintic treatment remains effective against *Ascaris* spp, reoccurring infections after treatment urge the need for a more permanent solution. Better knowledge of host-parasite interactions and the protective immune response should facilitate the development of potential vaccine candidates and might help explain epidemiological patterns.

*A. suum* has a complex life cycle, which starts when larvae hatch from ingested eggs. After penetrating the intestine at the caecum or proximal colon, L3 stage larvae migrate to the liver and subsequently to the lungs. Around 10 days post infection (DPI), the larvae are coughed up and ingested. Shortly after their arrival in the small intestine, the larvae molt to L4 stage. Between 14 and 21 DPI more than 95% of L4 larvae will be gradually eliminated from the small intestine, in what is known as the self-cure reaction or expulsion phase [6]. L4 stage larvae that survive past 28 DPI will grow into adults, preferentially inhabiting the proximal half of the small intestine. Pigs build up a strong protective immunity after a prolonged exposure to *Ascaris*. This protective immunity develops at the level of the gut and prevents the incoming larvae to penetrate the intestinal tissue and start their hepato-tracheal migration. This is the so-called pre-hepatic barrier [6]–[10]. Urban *et al.* elegantly demonstrated that the protective mechanism of this immune barrier was located at the level of the gut, as *in vitro* hatched larvae injected in the mesenteric veins caused white spots, while orally administered eggs did not [6]. Little is known of what immunological factors are associated with this protective immune mechanism. When initially the pre-hepatic barrier was described, it was still believed that larvae penetrated the small intestine. However, it was later discovered that in fact the caecum and proximal colon are the site of parasite entry [11]. The purpose of this study was therefore to identify the key immunological elements involved in the formation of the pre-hepatic barrier in the caecum of pigs following *Ascaris* infections.

## Materials and Methods

### Animals and parasites

All animal experiments were conducted in accordance with the E.U. Animal Welfare Directives and VICH Guidelines for Good Clinical Practice, and ethical approval to conduct the studies were obtained from the Ethical Committee of the Faculty of Veterinary Medicine, Ghent University. *A. suum* free, Rattlerow Seghers hybrid piglets of 10 weeks old were used. The animals had access to feed and water *ad libitum*.

*A. suum* eggs were obtained from gravid females collected at the local abattoir from pigs that were being processed as part of the normal work of the abattoir. After incubation in 0,1% KCr_2_ for 2 months, embryonation was confirmed by way of light microscopy.

For the *in vitro* tests, L3 stage larvae were collected from embryonated eggs. The eggs were incubated in sodium hypochloride for 1 h, washed with PBS and then hatched by magnetic stirring with 2 mm diameter glass beads. To separate the larvae from unhatched eggs, the suspension was put on a baermann sieve covered with cotton cloth. After overnight incubation at 37°C, the larvae were collected and put in DMEM medium supplemented with 50 u/ml penicillin, 50 µg/ml streptomycin, 100 µg/ml kanamycin, 5 µg/ml amphotericin B and 2 mM glutamine.

### Infection trial

The experimental design is summarized in Table 1. Three groups of pigs were used. A first group of six pigs were fed 100 *A. suum* eggs per day in a small food bolus for 14 weeks. Eggs per gram feces (EPG) were monitored weekly from week 6 onwards. After 14 weeks the animals were dewormed with fenbendazole (5 mg/kg). Two weeks after deworming, these animals received a first challenge infection of 5000 eggs. Thirteen days later, a second challenge infection of 5000 eggs was administered. Twenty-four hours later, the animals were euthanized for sample collection. These animals are referred to as immune animals. A second group of 5 naïve animals received anthelmintic treatment 2 weeks before being infected with 5000 eggs and euthanized 14 days post infection (DPI). These animals served to compare larval counts between immune and naïve animals at 14 DPI. A third group of 5 animals received anthelmintic treatment 2 weeks before being infected with 5000 eggs and euthanized 24 hours later to compare the early immune response with the immune animals that received a challenge infection 24 hours prior to necropsy.

**Table 1:** Infection protocol and worm counts.

Group	N^a^	Immunized^b^	Challenge 1^c^	Challenge 2^d^	Worm counts^e^
1	6	yes	yes	yes	8±4
2	5	no	yes	no	2333±496
3	5	no	no	yes	N.D.

a : number of animals in the group.

b : 100 *A. suum* eggs daily for 14 weeks.

c : 5000 *A. suum* eggs 14 days prior to necropsy.

d : 5000 *A. suum* eggs 24 hours prior to necropsy.

e : worm counts determined in the small intestine.

N.D. Not determined.

Animals were denied feed from 24 h before until necropsy and then killed with a captive bolt pistol, exsanguinated and the intestines were removed. Samples for RNA extraction and histological analysis were taken from the caecum. The small intestine was washed and the contents passed through a 220 µm sieve. *A. suum* larvae were counted under a microscope.

### RNA extraction, cDNA synthesis and real time PCR assays

Tissue samples from the caecum were taken from group 1 and 3 and immediately snap frozen in liquid nitrogen and stored at −80°C until RNA extraction. RNA extraction was performed using Trizol reagent (Invitrogen), combined with an RNeasy mini kit (Qiagen). A DNase treatment was included to prevent genomic contamination. RNA integrity was assessed using a Biorad Experion with a standard sensitivity chip. cDNA was synthesized with a Biorad cDNA synthesis kit, starting from 1 µg of RNA.

Primers for the real time PCR reactions were designed with the Primer3 software [12], or taken from the PIN database (http://199.133.11.115/fmi/iwp/cgi?-db=PINdb&-loadframes). For a list of primers, see Table S1. PCRs were run using Fast SYBR Green Master Mix (Applied Biosystems) on an AB StepOnePlus Real-Time PCR System. Primer specificity was confirmed by observing the melting curve. PCR products were confirmed through sequencing. Gene expression levels were normalized based on housekeeping genes selected using Genorm [13]. Housekeeping genes tested were: *b2m, gapdh, hmbs, rpl4, tbp1* and *ywhaz*. The genes selected for normalization were *hmbs* and *tbp1*. Gene transcription levels are expressed as fold change in transcription levels of immune animals compared to naïve animals.

### Histological analysis

Tissue samples were taken from animals in group 1 and 3 and were washed in PBS, processed with the Swiss roll technique [14] and fixed in either 10% formaldehyde or Carnoy's fixative for 24 h. After fixation, the tissues were dehydrated by passage through a series of graded alcohol dilutions, followed by embedment in paraffin. Tissue samples were cut in 4 µm sections. To assess general histopathological damage and the accumulation of eosinophils, formaldehyde fixed samples were routinely stained with haematoxylin-eosin. Mucosal eosinophils were counted at 400× magnification on 10 fields corresponding to 0,162 mm^2^. Mast cells were counted on toluidine blue stained slides at 200× magnification using a weibel2 graticule [15]. Goblet cells were counted on Alcian blue-periodic acid shiff's stain and expressed as number of goblet cells per 100 µm crypt length. For immunohistochemistry, formaldehyde fixed, paraffin embedded sections were rehydrated and an antigen retrieval step with citrate buffer was included. Endogenous peroxidase activity was blocked using 1% hydrogen peroxide. Sections were stained with mouse anti-human MAC387 (Serotec) to stain macrophages. Biotinylated secondary antibodies (Dakocytomation A/S) were added and staining was performed using the peroxidase streptavidine complex (Dakocytomation A/S), diaminobenzidine tetrahydrochloride (DAB, Sigma–Aldrich) and H_2_O_2_. Sections were subsequently counterstained with haematoxylin. Macrophages were counted at 200× magnification using a weibel2 graticule [15]

### Isolation of circulating eosinophils

Peripheral blood was collected on EDTA from the jugular vein of pigs at 14 DPI. The blood was diluted with an equal amount of PBS and layered onto a discontinuous Percoll gradient (68% and 75%) and centrifuged (500×*g* at 4°C for 30 min) to separate the granulocyte fraction. After lysis of contaminating erythrocytes in 0,2% NaCl solution, eosinophils were separated by negative magnetic activated cell separation with mouse anti-pig CD16 antibody (AbD Serotec) and rat anti-mouse IgG1 microbeads (Miltenyi-Biotec). The purity of eosinophils was verified with a Giemsa stain after cytospin and was >95%. The cells were washed three times and resuspended at 10^6^ cells/ml in RPMI-1640 without phenol-red.

### Eosinophil degranulation assay

The degranulation assay was essentially performed as described by Donne *et al.* [16]. Reactive oxygen species production was measured using a chemiluminescence assay. Eosinophils from 1 pig were seeded in a 96-well plate at 2×10^5^ cells/well in 200 µl of RPMI without phenol-red. The plates were incubated at 37°C for 2 h in a humidified atmosphere with 5% CO_2_, so that the cells could adhere to the plastic surface. The supernatant was removed and 100 µl luminol (1 mM) in HBSS with Ca^2+^/Mg^+^ was added. After 5 min of background measurement at 37°C, 100, 200 or 300 *A. suum* L3 larvae in HBSS were added in 100 µl as well as the control agents (PMA 5 µg/ml as positive control and HBSS with Ca^2+^/Mg^+^ as negative control). To test if there was antibody or complement dependent degranulation, serum taken either from 5 uninfected naive or 5 immune animals was pooled and added at 1/100 dilution. Heat inactivation of serum was done at 58°C for 30 minutes. ROS-production was measured during 120 min in the integration mode. Each condition was performed in triplicate and ROS-production was expressed as the fold change in relative light units (RLU) compared to negative controls (HBSS). The experiment was performed 3 times independent from each other.

### *A. suum* L3 viability assay

Eosinophils from 1 animal were seeded at 2.10^6^/ml in 100 µl in a 96 well plate in RPMI supplemented with 50 u/ml penicillin, 50 µg/ml streptomycin and 2 mM glutamine. L3 larvae were added at 100 per well, with or without serum pooled from 5 uninfected naïve or 5 immune pigs at a final concentration of 1/100. After 16 h of incubation, viability of L3 larvae was assessed morphologically. Curled up or moving larvae were considered alive, while immobile, straight larvae were considered dead. Viability was expressed as the number of live larvae to the total number of larvae. Every condition was assessed in triplicate with eosinophils from 2 different animals. Negative control conditions consisted of medium without eosinophils.

Viability was also tested using an MTT assay as previously described [17]. Briefly, eosinophils were seeded at 2.10^6^/ml in a 96 well plate in 100 µl RPMI supplemented with 50 u/ml penicillin, 50 µg/ml streptomycin and 2 mM glutamine. 100 L3 larvae were added per well with or without serum pooled from 5 naïve or 5 immune pigs at a final concentration of 1/100. MTT was added at a final concentration of 1 mg/ml. After 3 h of incubation at 37°C and 5% CO_2_, larvae were collected, washed and transferred to DMSO. After 1 hour the plate was read at 562 nm. Every condition was tested in triplicate.

### Statistical analysis

For statistical analysis, GraphPad Prism software (v5.0c) was used. Unpaired student t-tests were used to test differences between immune and naïve animals. The data collected from each group in the degranulation and viability assays were compared by analysis of variance (ANOVA) using the SPSS v20.0 software package.

## Results

### Parasitological data

The infection protocol and worm counts are summarized in Table 1. Pigs in group 1 were immunized for 14 weeks with 100 eggs/day. The average EPG at 14 weeks was 4008 (range 50-11050). The animals were dewormed and then challenged with 5000 eggs. Worm counts at 14 days post challenge were compared to naïve animals receiving only anthelmintic treatment and the challenge infection (group 2). Immune pigs had a 99,7% reduction in the number of larvae that can migrate through the body and reach the small intestine compared to naïve pigs from group 2 (8±4 in immune group versus 2333±496 in naïve group).

### Cellular parameters associated with immunity

**Figure 1:**
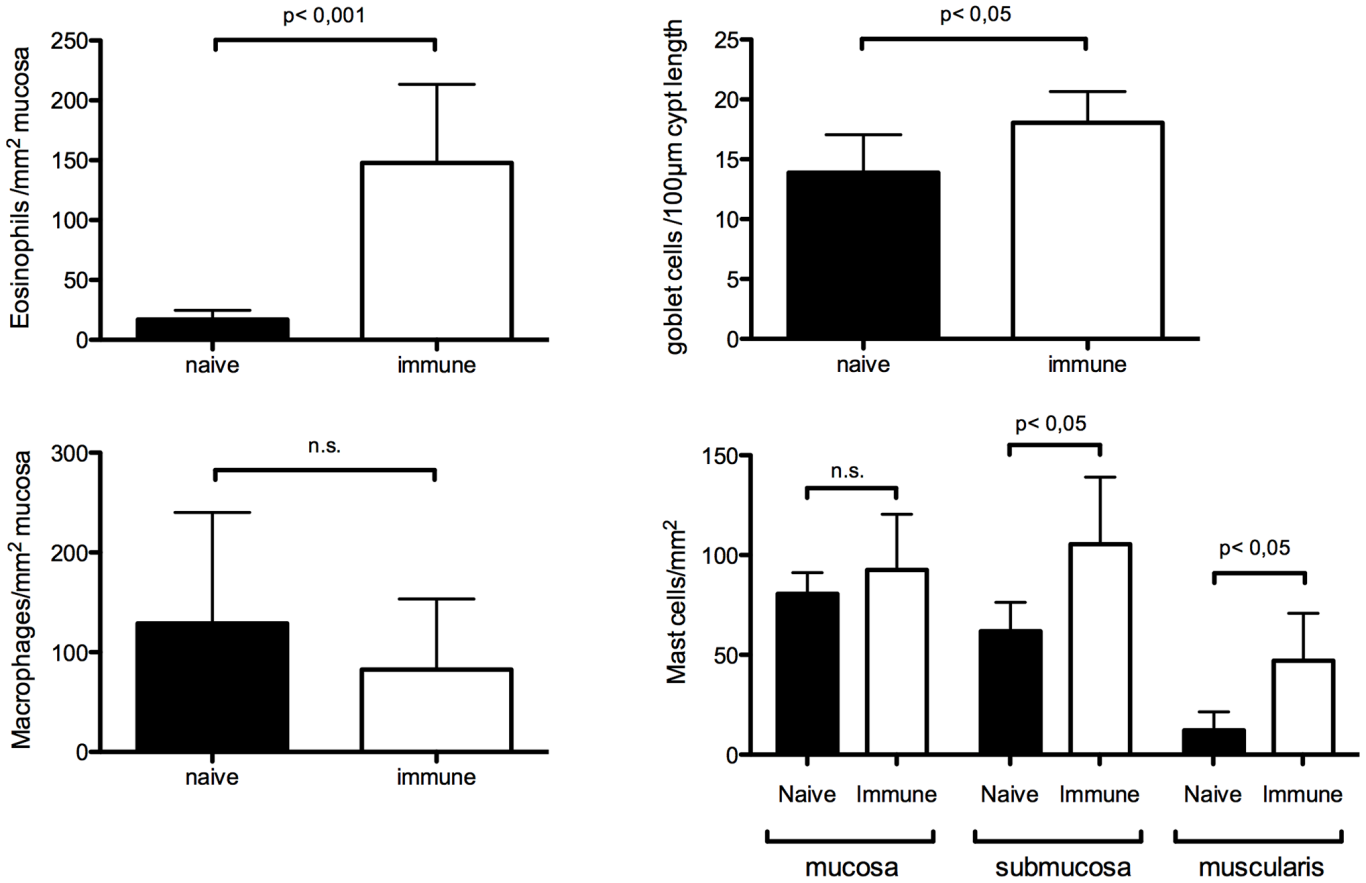
Eosinophil, macrophage, goblet cell and mast cell counts in the caecum of naïve and immune animals. Results are shown as average + SD. n.s.: not significant.

Caecal tissue was collected from naïve and immune animals 24 hours post challenge. Eosinophils, goblet cells, macrophages and mast cells were quantified and results are shown in Figure 1. The major effect was seen for eosinophils, with a significant almost 10-fold increase in mucosal eosinophils (p<0,001) in the immune animals. There was also a modest increase in goblet cells in immune animals (p<0,05). In addition, mast cells seemed to be specifically recruited to the submucosa and muscularis layers of the caecum (p<0,05) of immune animals. No significant difference was observed for the number of macrophages between naïve and immune animals.

### RNA transcription profile

The outcome of the qRT-PCR analyses is shown in Table 2. Significantly higher transcription levels for *c3* (complement factor 3), *ccl11* (Eotaxin), *ccr3*, *epx* (Eosinophil peroxidase), *gata3, il5, il12b, il13* and *retnlb* (Resistin Like Beta) were detected in the caecum of immune animals, whereas *muc5ac* (mucin 5AC) was significantly down regulated in immune animals compared to naïve ones. No significant differences were observed for the other genes analyzed.

**Table 2:** RNA transcription profile of the caecum.

Gene	Description	Fold change	
ARG1	Arginase I	0.65	
C3	Complement factor 3	1.84*	
CCL11	Chemokine (C-C motif) ligand 11, Eotaxin 1	2.50*	
CCR3	Chemokine (C-C motif) receptor 3, Eotaxin receptor	4.70*	
ELANE	Elastase, neutrophil expressed	0.89	
EPX	Eosinophil peroxidase	10.2*	
FOXP3	Forkhead box P3	1.05	
GATA3	GATA binding protein 3	1.62*	
IFNy	Interferon γ	1.27	
IL10	Interleukin 10	1.28	
IL12A	Interleukin 12 subunit p35	0.98	
IL12B	Interleukin 12 subunit p40	2.43*	
IL13	Interleukin 13	2.57*	
IL17A	Interleukin 17 A	1.87	
IL33	Interleukin 33	0.71	
IL4	Interleukin 4	1.12	
IL5	Interleukin 5	1.65*	
ITLN2	Intelectin 2	1.70	
MRC1	Mannose receptor C type 1	1.06	
MUC1	Mucin 1	1.36	
MUC2	Mucin 2	1.19	
MUC3	Mucin 3	1.08	
MUC5AC	Mucin 5 AC	0.17*	
RETNLB	Resistin-like molecule β	2.33*	
TGFB	Transforming growth factor β	0.96	
TNFA	Tumour necrosis factor α	1.16	

Results are shown as average fold change of transcription of immune animals versus naïve animals + SD.

* p<0.05.

### Eosinophil ROS production in response to *A. suum*

**Figure 2:**
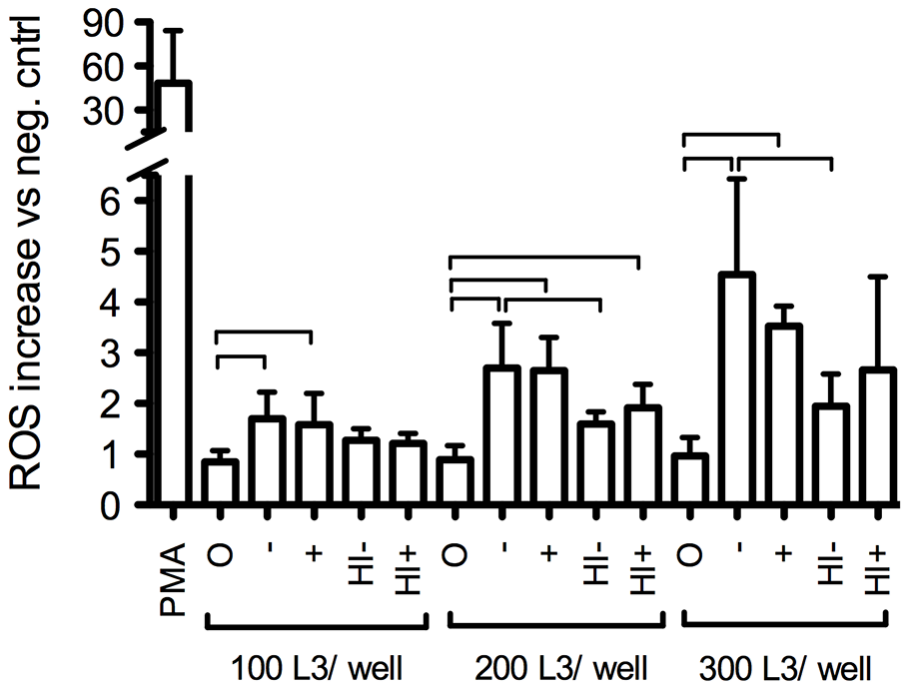
Eosinophil ROS production in response to direct contact with infective larvae. Eosinophils were purified from blood of animals at 14 DPI. 2.10^5^ eosinophils from 1 animal were seeded per well in HBSS. PMA: Phorbol myristate acetate (5 µg/ml), positive control. HBSS: negative control. O: no serum added. −: Serum pooled from 5 naive animals added. +: Serum pooled from 5 immune animals added. HI−: heat inactivated serum pooled from 5 naïve animals. HI+: heat inactivated serum pooled from 5 immune animals added. Results shown are expressed as the fold increase in ROS production compared to negative control (HBSS) and are the average + SD of 3 experiments with different animals. The bars indicate statistically significant differences between groups (p<0,05).

To investigate if eosinophils degranulated in the presence of infective L3 larvae, reactive oxygen species (ROS) release was measured in the medium for 2 hours following the addition of larvae to purified eosinophil cultures (Figure 2). Eosinophils or larvae alone with serum did not induce ROS release and eosinophils did not degranulate when larvae were added in the absence of serum. However, when serum from either immunized or naïve animals was added together with the L3 larvae, eosinophils released ROS in the medium. The release of ROS was proportional to the amount of larvae added. Heat-inactivation of serum reduced the amount of ROS release.

### Viability of infective *A. suum* larvae after culture with eosinophils

**Figure 3:**
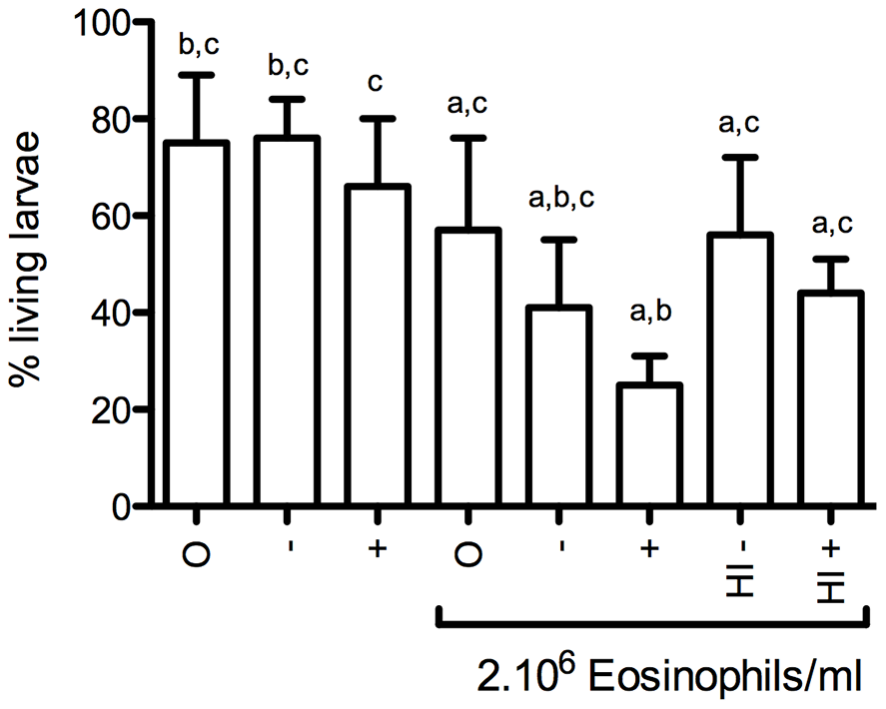
*A. suum* L3 larvae viability after culture with eosinophils. Eosinophils were purified from blood of animals at 14 DPI. Viability was assessed visually after 16 hours of incubation with 100 L3 larvae. O: no serum added. −: Serum pooled from 5 naive animals added. +: Serum pooled from 5 immune animals added. HI−: heat inactivated serum pooled from 5 naïve animals. HI+: heat inactivated serum pooled from 5 immune animals added. Results are shown as mean + SD of two independent experiments with three incubations each. a: significantly different than L3 cultured without eosinophils or serum (p<0,05). b: significantly different than L3 cultured with eosinophils without serum (p<0,05). c: significantly different than L3 cultured with eosinophils and serum from immune animals (p<0,05).

**Figure 4:**
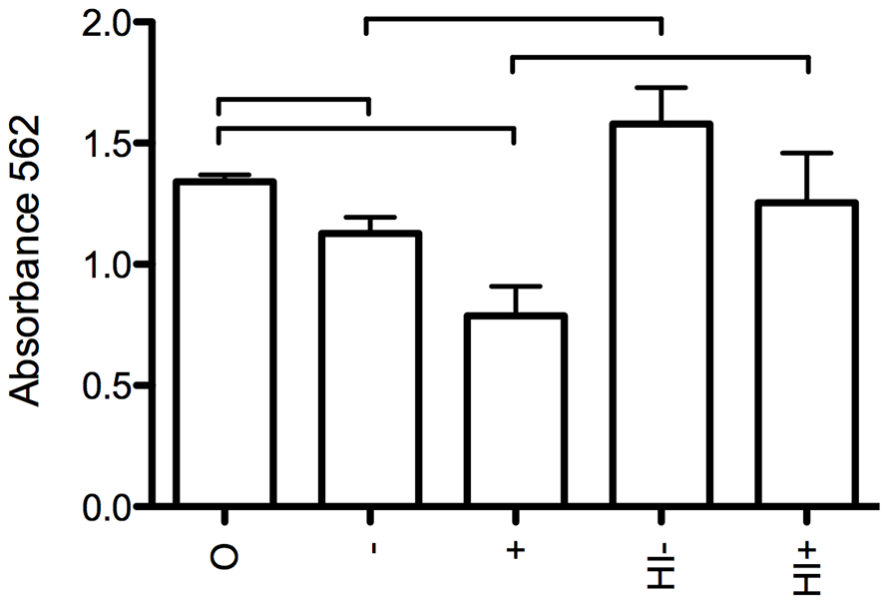
MTT assay of viability of infective larvae after culture with eosinophils. Eosinophils were purified from blood of 1 animal at 14 DPI. 2.10^6^/ml eosinophils were incubated together with 100 infective L3 *A. suum* larvae. Viability was determined by the MTT assay after 3 hours of incubation. O: no serum added. −: Serum pooled from 5 naive animals added. +: Serum pooled from 5 immune animals added. HI−: heat inactivated serum pooled from 5 naïve animals. HI+: heat inactivated serum pooled from 5 immune animals added. Results are shown as mean + SD of three incubations. The bars indicate statistically significant differences between groups (p<0,05).

Eosinophils were cultured together with infective L3 stage *A. suum* larvae for 16 hours after which viability of the larvae was assessed (Figure 3). Eosinophils had a toxic effect on the L3 larvae, which was enhanced when serum from naive animals was added and was highest when serum from immune animals was added. Heat inactivation of serum led to reduced killing compared to non-heat inactivated serum. Similar results were obtained with the MTT colorimetric assay (Figure 4).

## Discussion

In this study we showed that pigs continually exposed to infective *A. suum* eggs for 14 weeks developed an almost sterilizing immunity, demonstrated by a 99,7% reduction in number of larvae that were able to migrate through the host, and that this immunity was associated with eosinophilia, mastocytosis and goblet cell hyperplasia in the caecum. To our knowledge, this is the first study to describe the immunological parameters at the actual site of parasite penetration, i.e. the caecum or proximal colon. Although immunity against *A. suum* infections can occur at the different organs affected, Urban *et al.* showed that the strongest response is already at the level of the gut [6]. They reported increased mast cell and eosinophil numbers in the small intestines of animals with intestinal immunity to *A. suum*. However, since it was later discovered that in fact the caecum and proximal colon are the site of parasite entry, it was unclear whether these findings reflected the response against the adult worms residing in the small intestine, rather than the response against the invading larvae.

In our experiments, only a few larvae could complete their migration and reenter the small intestine. These few larvae would have a minimal impact on the immunological parameters observed in the caecum, since protective immunity was already present at the time of first challenge and results from another experimental infection trial performed by our research group showed that the presence of approximately 50 L4's in the small intestine at 14 DPI in a primary infection did not result in eosinophilia, mastocytosis or goblet cell hyperplasia in the caecal tissue (unpublished observations). Furthermore, it was previously shown that removal of adult *A. suum* worms before challenge did not influence immunity against invading larvae [7].

We observed an almost 10-fold increase in mucosal eosinophils in immune animals. The recruitment of eosinophils to the caecum of immune animals was further supported by increased levels of IL-5, IL-13, CCL11 and eosinophil peroxidase (EPX) transcripts in the caecal mucosa. IL-5 is one of the key cytokines involved in the development of eosinophils. It is also essential in the recruitment of eosinophils from the bone marrow to the blood [18]. CCL11, also termed Eotaxin 1, is an eosinophil specific chemoattractant and functions to home eosinophils from blood to tissue and it can be induced by IL-13 [18]. EPX is a granule protein specific for eosinophils and results in the formation of reactive oxygen species [19]. As the *A. suum* larvae penetrate the caecal mucosa to reach the liver, they are likely to come into close contact with the mucosal eosinophils. Circulating eosinophils responded *in vitro* to direct contact with the larvae by releasing the contents of their granules. This degranulation was observed with serum from both infected and uninfected animals and the effect was diminished when serum samples were heat-inactivated, indicating that at least a part of it was complement dependent. *A. suum* specific antibodies appear to be non-essential in the degranulation, since serum from immune animals did not lead to increased degranulation compared to serum from naïve animals. However, it is important to note that the experiments were performed with circulating eosinophils and so it still has to be determined to what extent mucosal eosinophils would respond similarly.

Previous work with guinea pigs and mice has shown that complement components can bind the surface of the *Ascaris* larvae and that leukocytes may damage larvae in the presence of serum [20], [21]. In the current study, we extended this knowledge by demonstrating that the combination of purified circulating eosinophils from the natural host and serum from immune animals was highly effective in killing the infective larvae. Since the most efficient killing of the larvae was in the presence of serum from immune animals, *A. suum* specific antibodies, in addition to complement components, probably also play an important role in the toxicity towards the parasite. In humans, IgG and IgE are the predominant isotypes for the killing of schistosomula by eosinophils [22], [23]. Although we did not test isotype specific responses, these isotypes might also be involved in the *Ascaris* larval killing, since these *A. suum* specific antibody isotypes were elevated from 5–6 weeks of exposure to *A. suum* eggs (data not shown).

Eosinophils have long been associated with helminth infections and antibody dependent eosinophil cytotoxicity against helminths *in vitro* was first shown for *Shistosoma* [23]. Toxicity of eosinophil granule proteins against nematodes has been shown for *Toxocara canis*, *Trichinella spiralis*, and *Brugia malayi*, mostly against juvenile stages [19]. Indeed, eosinophils appear to be essential only in the defense against juvenile, tissue-residing helminthes [24]. Our findings support this conclusion, as eosinophils only degranulated in response to the tissue dwelling L3 larvae, and not the lumen dwelling L4 larvae (unpublished data). It would be interesting to investigate if these differences are caused by diminished complement activation in different life stages of *Ascaris*, as is the case for *Nippostrongylus brasiliensis* [25]. To build up a high enough concentration of eosinophils, complement and antibodies at the site of parasite entry probably requires multiple infection cycles over a longer period of time. This would explain why sterilizing immunity is not established until after several weeks of exposure to infectious *A. suum* eggs.

In addition to eosinophils, mast cells were also recruited to the submucosa and muscularis layer of the caecum of the immune animals. Whether or not mast cell derived products have direct effects on the invading larvae is unclear, but their submucosal and muscularis location would suggest that mast cells would more likely act in an indirect manner. Mast cells add to the general inflammation by producing Th2 type cytokines such as IL-4, IL-5 and IL-13. They are also the primary source of histamine. It was previously shown that mast cells and basophils from repeatedly infected animals released histamine after contact with *Ascaris* secretory antigens [26], [27]. Histamine has various functions. Amongst others, it works as a chemoattractant for eosinophils and histamine release by mast cells can also induce smooth muscle contractions [28]. Additionally, mast cell proteases can break tight junctions, leading to increased intestinal fluid secretion. Although we did not measure fluid secretion and muscle contractions, they are part of a ‘weep and sweep’ response that is often seen in gastro-intestinal infections [29] and might contribute to the resistance against *Ascaris*.

Interestingly, Urban *et al.* previously also described eosinophilia and mast cell influx in the midgut region of the small intestine of animals with a pre-hepatic barrier [7]. Whether the influx of these immune cells is a result of the development of the pre-hepatic barrier at the level of the caecum and colon or rather caused by the exposure of the small intestinal mucosa to L4 stage larvae and adults worms is still unclear.

We also identified goblet cell hyperplasia in animals resistant to invading *Ascaris* larvae. Increased mucus production is often part of a general Th2 type response against gastro-intestinal nematode infections [30]. It might play an important role as it could trap the hatched larvae, making it more difficult to penetrate the intestinal wall. Despite the apparent goblet cell hyperplasia, we could not demonstrate an increase in any specific mucin on transcriptional level. Although mucin 5AC has been described as a crucial mucin in the expulsion of gastro-intestinal nematodes in rodent models [31] and is up regulated in pigs infected with *Trichuris suis* [32], *muc5ac* was significantly down regulated in immune pigs compared to naïve ones. The apparent down regulation of *muc5ac* in immune animals may however reflect an early increase in transcription caused by the challenge infection in the naïve animals. In addition to mucus production, goblet cells also secrete proteins with antimicrobial properties. We demonstrated a significant increase in transcription of *retnlb*, the gene coding for Relmß. This goblet cell specific protein has shown to have direct anthelmintic properties. Relmß knockout mice are more susceptible to *N. brasiliensis* and *Heligmosomoides polygyrus* [33] and it was also shown that Relmß was able to bind the lateral alae of *Strongyloides stercoralis*, thereby disrupting chemotactic functions [34]. Whether it acts in a similar way against *A. suum* is still unclear and needs further research.

It is unclear to what extent the results obtained with *A. suum* in pigs can be extrapolated to humans and *A. lumbricoides*. However, similar infection patterns are observed in humans and because of the extremely high similarity between these two parasites on molecular level, there is even question whether or not *A. suum* and *A. lumbricoides* are the same species [35], [36]. Eosinophilia is also often observed in humans infected with *A. lumbricoides*, but the link with protection against reinfection has not been made. Nevertheless, it seems likely that in humans eosinophils also play a crucial role in the defense against invading larvae, as pre-treatment levels of IL-5 in humans are also related to resistance against reinfections with *A. lumbricoides* [37]. The fact that immunity against *Ascaris* is only built up after continuous exposure over a long period of time might explain why reinfections are so common in children treated for *Ascaris*. However, it is also likely that as the immune response increases with exposure, fewer larvae will be able to penetrate the gut and as such acute morbidity due to the hepato-tracheal migration will be lower as children age.

In conclusion our results indicate that mast cells, eosinophils and goblet cells operate together to create an inhospitable environment that protects the host against invading *Ascaris* larvae. A general Th2 response, propagated by mast cells and eosinophils seems pivotal in the resistance against invading larvae. The current focus of our research is in identifying which antigens are targeted in immune animals and are essential in eosinophil degranulation in different life stages of *A. suum*, in an effort to find suitable targets for vaccine development.

## Supporting Information

Table S1 **Primer sequences.**
(DOCX)
