# Supplementary material for: A Role for Eosinophils in the Intestinal Immunity against Infective Ascaris suum Larvae
Source: PLoS Negl Trop Dis. 2013 Mar 21;7(3):e2138. doi: 10.1371/journal.pntd.0002138 (PMC3605247; doi:10.1371/journal.pntd.0002138)
Supplement: Table S1 — Primer sequences. (DOCX) [file pntd.0002138.s001.docx]

**Table S1**

| **Gene** | **Forward primer** | **Reverse primer** | **Accesion number** |
| --- | --- | --- | --- |
| ARG1 | GGCCACTGGCACACCAGTCC | ACTGCCGTGTTCACCGTCCG | NM_214048.2 |
| B2M | CACTCCTAACGCTGTGGATCAG | CCACTTAACTATCTTGGGCTTATCG | AB436775.1 |
| C3 | CAAGAAATGATTGGTGGCTTCAA | GACCTGTGGTTCACAGATGTCTTT | NM_214009 |
| CCL11 | CTTCTGTCGCCACCATCTG | ATTCTCTTGGGCATCAGCAC | XM_003131725.1 |
| CCL2 | GCGGCTGATGAGCTACAGAAG | CCGCGATGGTCTTGAAGATC | NM_214214 |
| CCR3 | ACAATGTTCTGCATCTGACCTAAAAT | AGAATGGAAAGAACCAGCTCTGTCT | NM_001001620 |
| ELANE | CAGCTCAACAGATTTGCCTTCA | ACGCCTTGGTCCTGAGCA | FP015903.2 |
| EPX | TGGCCTCCCAGGGTACAAT | CAGGAACTTCCTCGCCAAAG | Ssc.33169 |
| FOXP3 | GGTGCAGTCTCTGGAACAAC | GGTGCCAGTGGCTACAATAC | AY669812 |
| GAPDH | GGCATGGCCTTCCGTGT | GCCCAGGATGCCCTTGAG | DQ845173.1 |
| GATA3 | TCTAGCAAATCCAAAAAGTGCAAA | GGGTTGAACGAGCTGCTCTT | NM_001044567 |
| HMBS | GCACGGCCATGTCTGGTAAC | CCACCACACTGTCCGTTTGTAT | NM_001097412 |
| IFNG | TGGTAGCTCTGGGAAACTGAATG | GGCTTTGCGCTGGATCTG | AY188090 |
| IL10 | TGAGAACAGCTGCATCCACTTC | TCTGGTCCTTCGTTTGAAAGAAA | NM_214041 |
| IL12A | GGCCTGCTTACCACTTGAAC | GCATTCATGGCCTGGAACTC | NM_213993 |
| IL12B | CTGAAGAAGACGGCATCACG | AGGAGTGACTGGCTCAGAAC | NM_214013 |
| IL13 | CTGACCACCAGCATGCAGTACT | GCTGCAGTCGGAGATGTTGA | NM_213803 |
| IL17A | CCAGACGGCCCTCAGATTAC | CACTTGGCCTCCCAGATCAC | NM_001005729.1 |
| IL33 | AGCTTCGCTCTGGCCTTATC | GCTGACAGGCAGCAAGTACC | XM_003121912.1 |
| IL4 | GCCGGGCCTCGACTGT | TCCGCTCAGGAGGCTCTTC | NM_214123 |
| IL5 | TGGTGGCAGAGACCTTGACA | CCATCGCCTATCAGCAGAGTT | AJ010088 |
| ITLN2 | CCTATGGACAAAGGGAATTTGTG | GCAGCCGGTAACTCTCATCC | NM_001128453 |
| MRC1 | GGATGGCTCTGGTGTGGAA | AATGCTGGTCAGTGGATCTTTATTC | AY368183 |
| MUC1 | GTGGGCAGCTGGACATCTTT | GCCTGCAGAAACCTGCTCAT | NC_010446 |
| MUC2 | GTGCAGGTGCAGGTCAACA | AGAGGCCGTTGTAGGAGATGAG | BX671371 |
| MUC3 | AGTGGTTCGAGATCTGGGATGA | CCAAGGCCACATGGAGGTT | BP153612 |
| MUC5AC | TGCTCCTGGTCCAAGTGGTT | GGAGGATATTGCTGTAGGTCTCAAA | AF054583 |
| RETNLB | GCCTTTCTATAGGATGAAGCCAACT | ACAAGGGAGTCTAAGGAACACTGAGA | NM_001103210 |
| RORC | AGCCCAGTCGGCAAAGC | TTGTCCCCACAGATTTTGCA | XM_001929621.1 |
| RPL4 | CAAGAGTAACTACAACCTTC | GAACTCTACGATGAATCTTC | DQ845176.1 |
| TBP1 | AACAGTTCAGTAGTTATGAGCCAGA | AGATGTTCTCAAACGCTTCG | DQ178129 |
| TGFb | GAAGCGCATCGAGGCCATTC | GGCTCCGGTTCGACACTTTC | NM_214015 |
| TNFa | CCAATGGCAGAGTGGGTATG | TGAAGAGGACCTGGGAGTAG | X54859 |
| YWHAZ | ATGCAACCAACACATCCTATC | GCATTATTAGCGTGCTGTCTT | XM_001927228.2 |
